# Plasmonic Characterization of 3D Printable Metal–Polymer Nanocomposites

**DOI:** 10.1021/acsmaterialsau.4c00007

**Published:** 2024-05-15

**Authors:** María de la Mata, Albeto Sanz de León, Luisa M. Valencia-Liñán, Sergio I. Molina

**Affiliations:** Departamento de Ciencia de los Materiales, I. M. y Q. I., IMEYMAT, Universidad de Cádiz, Campus Rio San Pedro, 11510 Puerto Real, Spain

**Keywords:** localized surface plasmon resonances, metal−polymer nanocomposites, electron energy loss spectroscopy, UV–vis absorbance, far- and near-field performance

## Abstract

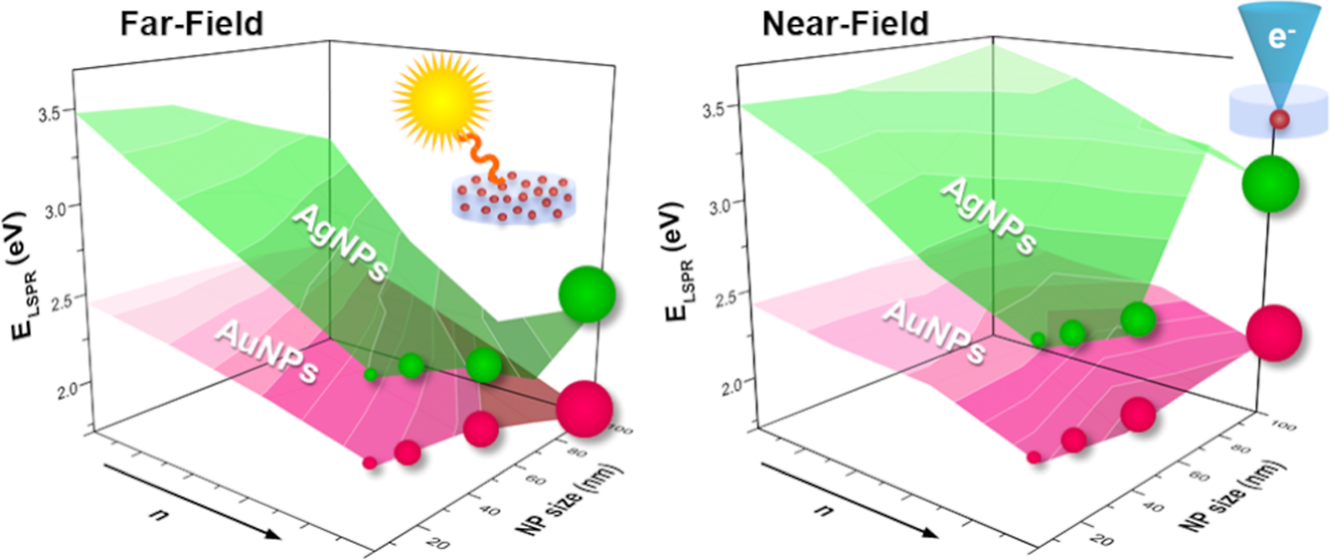

Plasmonic polymer nanocomposites (i.e., polymer matrices containing plasmonic nanostructures) are appealing candidates for the development of manifold technological devices relying on light–matter interactions, provided that they have inherent properties and processing capabilities. The smart development of plasmonic nanocomposites requires in-depth optical analyses proving the material performance, along with correlative studies guiding the synthesis of tailored materials. Importantly, plasmon resonances emerging from metal nanoparticles affect the macroscopic optical response of the nanocomposite, leading to far- and near-field perturbations useful to address the optical activity of the material. We analyze the plasmonic behavior of two nanocomposites suitable for 3D printing, based on acrylic resin matrices loaded with Au or Ag nanoparticles. We compare experimental and computed UV–vis macroscopic spectra (far-field) with single-particle electron energy loss spectroscopy (EELS) analyses (near-field). We extended the calculations of Au and Ag plasmon-related resonances over different environments and nanoparticle sizes. Discrepancies between UV–vis and EELS are dependent on the interplay between the metal considered, the surrounding media, and the size of the nanoparticles. The study allows comparing in detail the plasmonic performance of Au- and Ag-polymer nanocomposites, whose plasmonic response is better addressed, accounting for their intended applications (i.e., whether they rely on far- or near-field interactions).

## Introduction

Plasmonic nanocomposites based on polymer matrices containing metal nanoparticles (NPs) have enormous potential as working materials intended for widespread applications.^1^ Importantly, polymer-based composites meet the advantages of polymer matrices and the functionalities of the hosted metal NPs. Therefore, the incorporation of suitable NPs into polymers provides plasmonic activity to the composite,^2^ while the overall mechanical behavior of the polymer is mostly preserved. Polymer matrices render, among other advantages, lightness and flexibility and allow for alternative 3D printing processing routes, such as stereolithography (SL). Remarkably, SL enables shaping the material at will, leading to final pieces and devices made from the target material,^3^ expanding exponentially the practical uses of the composites. Indeed, SL allows the fabrication of polymer or polymer-based composites with higher resolution than other 3D printing techniques, reaching values down to a few microns.^4^ This paves the way toward the development of tailor-made objects with complex geometries that cannot be manufactured by other technologies, satisfying the continuously growing demand of different industrial sectors in the field of nanotechnology. Within this context, it is essential to have a deep understanding of the spectral response of the materials to fine-tune their performance in order to meet the requirements for certain applications. Remarkably, 3D printing offers a powerful tool for processing optic and photonic devices,^5,6^ enabling, for instance, manufacturing lenses for multiple purposes (imaging, detection, etc.) or optical filters intended, for example, for colorblindness correction.^7^

The utility of plasmonic nanostructures stems from their ability to enhance light–matter interactions,^8^ rendering appealing systems for countless applications,^9,10^ such as energy harvesting, photocatalysis,^11^ imaging,^12^ sensing,^13^ etc. Surface plasmon resonances are collective electron oscillations created between two materials with opposite signs in the real parts of their dielectric permittivity.^14^ The plasmon resonance becomes strongly localized at the surfaces of nanostructures, resulting in the so-called localized surface plasmon resonances (LSPRs). In particular, LSPRs from noble metal NPs, such as gold, Au, or silver, Ag, show outstanding plasmonic responses within the visible (VIS) and near-infrared (IR) spectral ranges, suitable for surface enhanced Raman spectroscopy (SERS) applications^15−17^ or for the active control of color generation in electronic devices^18^ and also when hosted within polymer matrices.^19,20^ Other materials different than noble metals achieve plasmon resonances deeper within the IR (e.g., semiconductor nanostructures)^21^ or UV (e.g., aluminum, gallium) regions,^22^ spreading the application range and capabilities of plasmonic devices. The smart design of functional nanocomposites requires in-depth correlative studies. The optical properties of the matrices become strongly important since they provide the propagation media for the resonances. Indeed, changing the environment around the functional NPs not only shifts the resonant frequency but may also alter the spectral shape,^23^ as it has been reported, for instance, for substrate-supported nanostructures.^24,25^ Plasmonic polymer nanocomposites can be successfully obtained either by loading polymer matrices with metal NPs (ex situ created) or by means of in situ formation of NPs within the polymer from proper metal precursors.^2,19^ The functional response of these nanocomposites depends not only on the phases composing the hybrid system but also on the filler morphology^26,27^ (size and shape) and the loading content^28^ and its distribution.^17^ The actual shape of the nanostructures plays a chief role in the plasmonic behavior. Whereas the spectral response of small nanospheres is dominated by dipolar excitation, other nanoparticle shapes may involve a variety of resonant modes. For instance, elongated or 1D-like nanostructures, such as nanorods and nanowires, show differentiated resonances associated with transverse and longitudinal modes.^29,30^ More complex shapes (for example, nanocubes^31^ or nanostars^32^) lead to enriched spectra,^33^ with several multiple resonant modes associated with tips, corners, edges or surfaces. Hence, the ability to tune the nanocomposite microstructure at will opens the way to customize the spectral response qualitatively and quantitatively.

The overall optical properties of plasmonic composites can be easily addressed by means of UV–vis spectroscopy, providing the average response of the whole material from macroscopic far-field measurements. The measurements rely on light absorbance; thus, the experiment takes place upon photon excitation. While the experimental results are usually successfully explained attending to Mie theory, a deeper understanding of the plasmonic behavior is required from single-particle analyses,^34^ attainable by electron microscopy techniques. The polaritonic nature of the plasmons makes possible their excitation by photons and electrons so that the electronic excitation of plasmon resonances can be achieved with electron microscopes. In particular, scanning transmission electron microscopy–electron energy loss spectroscopy (STEM–EELS) enables the detection of differentiated resonant modes within individual nanostructures.^35,36^ The technique measures inelastic scattered electrons resulting from the interaction of the scanning electron probe with outer atomic electron shells, which are related to the optoelectronic behavior of the material. Therefore, in contrast to UV–vis spectroscopy, the EELS provides near-field measurements upon electron excitation. Moreover, the extraordinary combination of spatial and spectral resolution attainable by STEM allows 2D^37,38^ and 3D^24,39^ single-particle studies (maps and tomographic reconstructions), higher order multipole excitation, and the ability to probe dark resonant modes as well as bright modes.^24,40^ Consequently, plasmonic NPs have been broadly studied by means of EELS over the last years,^41^ including substrate-supported systems.^23^ EELS analysis becomes particularly useful to correlate the LSPR and local vicinity around plasmonic emitters within nanocomposites, proving, for instance, the influence of the hosting material or coupling between close neighboring NPs.^42^

Both, far- and near-field phenomena may contribute until different extents to the final response of the plasmonic system with diverse implications. In fact, the plasmonic response is usually a consequence of the interplay between the two effects, whose contribution depends strongly on the morphology and nature of the plasmonic emitter.^43^ For instance, the size of the NPs may allow tuning the contribution from the far-field mechanism to the plasmonic response since light scattering (far-field) is favored for larger NPs.^44^ While few applications rely either on far- or near-field phenomena (for example, sensors based on light scattering or near-field enhancement^19,45^), most plasmonic devices combine both contributions. Therefore, far- and near-field should be evaluated to provide a comprehensive full description of plasmonic systems.^46^ On one hand, the ability of plasmonic NPs to scatter incident photons at the far-field renders efficient systems for light trapping purposes (i.e., light harvesting).^43^ On the other hand, the near-field enhancement in the close vicinity of the NPs can be exploited, for instance, for the development of SERS sensors.^19^ Some applications involving both mechanisms include, among others, solar cells and photovoltaic devices.^43^

Most reported studies on the development of plasmonic nanocomposites focus on the control over the size distribution, the amount, and dispersion of the NPs, which are remarkably important but are not the unique factors dictating the plasmonic properties. Possible discrepancies between far- and near-field responses at polymer nanocomposites are usually neglected, hindering their accurate functional characterization. Importantly, properly addressing the material behavior enables fine-tuning its response to a greater extent, allowing the development of nanocomposites with highly customized properties. Focusing on 3D printable polymers, the ability to shape them at will spreads incommensurately their application range, whereas achieving the requested performance may rely on tailoring the functional response of the printed material. We analyze the optical activity of two plasmonic acrylic resin-based nanocomposites suitable for SL, with either Au or Ag NPs, by UV–vis spectroscopy and low-loss EELS. Thus, we provide macroscopic measurements along with single-particle measurements, including LSPR maps from individual NPs within the resin. We discuss the role of the metal (i.e., Au, Ag), the environment (i.e., air, acrylic resin), and the technique employed (i.e., UV–vis, EELS) on the plasmon energy and spectral shape, by comparing the experimental results with simulations. For the systematic comparison between far- and near-field responses, it is important to adequately use the optical characterization tools depending on the target application for the nanocomposites. The obtained insights not only prove the plasmon activity of the actual nanocomposites but also provide a comprehensive framework for further tuning their response between 2.3 and 3 eV.

## Results and Discussion

### Addressing Plasmonic Properties of Au- and Ag-Acrylic Resin Nanocomposites

We deeply characterize the optical response of two different types of plasmonic nanocomposites for SL containing either Au or Ag NPs. Both nanocomposites are synthesized following in situ approaches involving NP formation within the polymer matrix from inorganic salt precursors through different mechanisms. Au NPs are created after polymerization (conventional curing or 3D printing) of the acrylic composites containing Au^3+^ species by subsequent thermal treatment,^47^ while Ag NPs grow during the curing/printing process of the acrylic resin containing Ag^+^ upon UV irradiation.^48^

**Figure 1:**
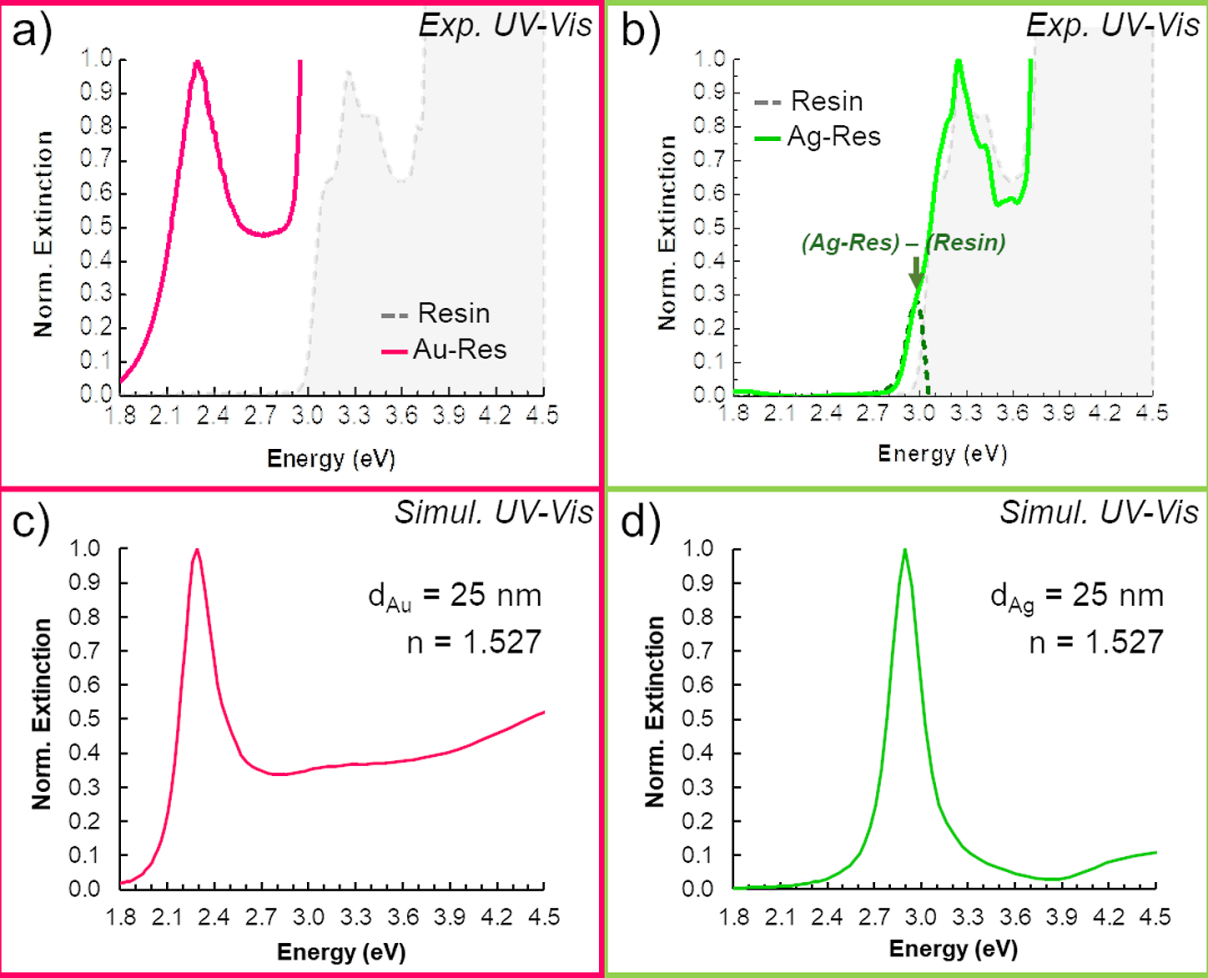
UV–vis (absorbance) spectra measured from Au- (a) and Ag- (b) nanocomposites, including the experimental spectrum from the pristine acrylic resin (dashed gray line). Au (c) and Ag (d) simulated extinction cross-section for NPs embedded in a medium with *n* = 1.527.

We address the optical properties of Au- and Ag-plasmonic nanocomposites by means of UV–vis spectroscopy, providing macroscopic results relying on far-field measurements. Figure 1a,b shows experimental UV–vis spectra from Au and Ag composites, respectively. We have replaced the typical wavelength by the corresponding energy in eV at the abscissa for ease of comparison with the EELS results. Importantly, both phases conforming the nanocomposites, i.e., the metal NPs and the resin, contribute to the spectral signal. The UV–vis spectrum from the pristine resin is included in both cases (dotted gray line), evidencing the signal overlap of NP and resin at Ag-nanocomposites. Such overlapping damps the Ag-related plasmon peak, which still can be inferred as centered at about 3 eV by accounting for the experimental spectral shape of the resin and the calculated Ag UV–vis signal (Figure 1d). In contrast, the lower characteristic resonant energy of Au NPs allows the accurate UV–vis determination of plasmonic activity at the Au nanocomposite, showing a clear peak center at 2.3 eV. The result is in good agreement with the presence of Au NPs with 25 nm diameter embedded in a medium with a refractive index of 1.527,^49^ corresponding to the acrylic resin value (Figure 1c). It is worth mentioning that we have assumed monodispersed spherical NPs for ease of calculations, even though wider size distributions are expected, and the NPs might not be perfect spheres, consequently broadening the experimental spectral lines. The effect of the size and refractive index of the matrix on the plasmonic response of Au and Ag NPs is discussed in greater detail in the following.

**Figure 2:**
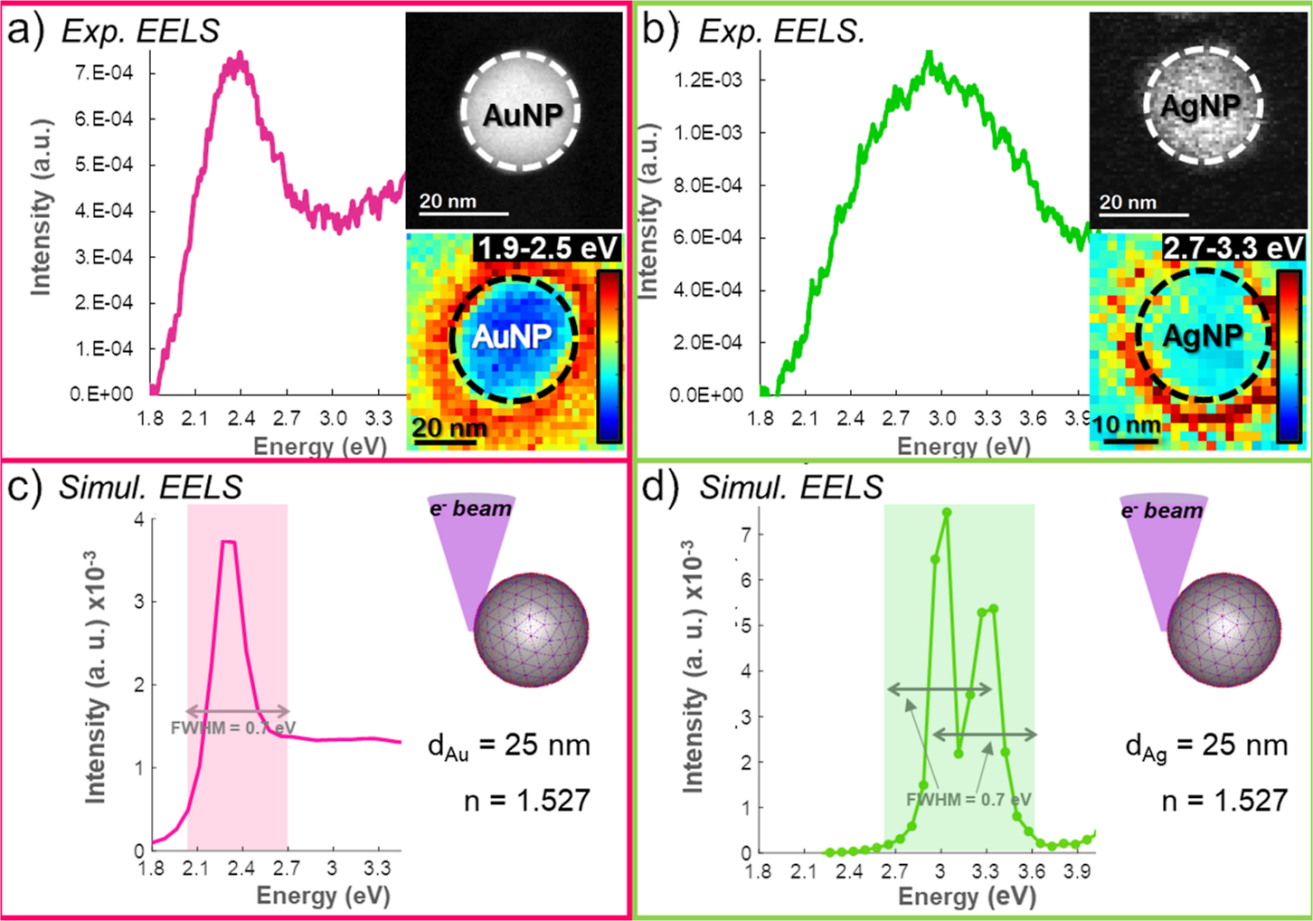
Experimental EEL spectra showing LSPR from single Au (a) and Ag (b) NPs inside the acrylic resin matrix and simulated EELS signals (c) and (d) for Au and Ag, respectively. The insets in panels (a, b) display STEM images of individual NPs within the acrylic resin (top) along with the EELS intensity maps (bottom) at 1.9–2.5 eV and 2.7–3.3 eV corresponding to LSPR from Au and Ag, respectively (intensity color scale superimposed).The insets in panels (c, d) depict the simulated systems.

We measured the LSPR from individual Au and Ag NPs (single-particle studies) embedded in acrylic resin nanocomposites by means of EELS. EELS analyses rely on near-field measurements performed upon electron excitation. Figure 2 shows experimental spectra from both plasmonic nanocomposites, containing Au (magenta) and Ag (green) NPs, acquired at the surface of the NPs, along with simulated EELS spectra (b,c). The simulations take into account the experimental diameter of the NPs measured from the STEM images acquired during the EELS experiments (see the insets in Figure 2a,b), the electron probe position, also referred to as impact parameter (*b*), and the refractive index (*n*) of the acrylic resin.

Regarding the experimental EEL spectral shape, the measured peaks are broader than the corresponding simulated signals, as expected from the experimental energy resolution achieved during the measurements (i.e., 0.7 eV). Moreover, the exact shape and actual environment surrounding the NPs directly affect the LSPR and, therefore, subtle deviation from the ideal system might contribute to the spectral broadening. Ag-related peak appears broader than that for Au, likely due to the spectral overlapping between the different contributions from the Ag plasmon and the polymer modes (evident at the UV–vis measurements, see Figure 1b), which may enable the energy transfer from NP to resin modes, driving the spectral line width broadening.^50^ Additionally, the plasmonic density of states of perfect Ag nanospheres is dominated by one broad band resulting from the superposition of high order polar modes only about 0.3 eV apart from the dipole mode.^24^ Indeed, the calculated EEL spectrum for a Ag NP of 25 nm in diameter placed in a medium with *n* = 1.527 shows two peaks just 0.31 eV apart, centered at 3.04 and 3.35 eV (Figure 2d), whose overlapping along with the experimental energy resolution may contribute to the peak broadening. The superposition of high order polar modes is also predicted for Ag NPs with similar dimensions embedded within SiO_2_ (*n* ≈ 1.5),^23^ leading to broader EEL plasmon-related peaks. Moreover, the reported experimental analysis on such material system evidences high-order multipole excitation at the surface of Ag NPs favored by the SiO_2_ matrix^51^ and overall spectral shapes in good agreement with our results. In contrast, LSPRs from Au NPs do not overlap the spectral response from the acrylic resin, and high-order mode contributions are not expected for 25 nm Au NPs (Figure 2c), resulting in narrower spectral lines (comparable to the electron beam size, i.e., to the fwhm of the transmitted electron beam, also called the zero-loss peak).

Importantly, plasmon near-field enhancement may distort meaningfully the spatial field distribution and its intensity in the vicinity of the NPs.^52^ The high spatial resolution and hyperspectral capabilities of STEM techniques, providing EEL spectrum imaging performance (i.e., sequential collection of EEL spectra over the scanning area), allow getting the near-field response at the nanoscale. Thus, in addition to getting local EEL spectra from individual Au and Ag NPs, we map the LSPR intensity from the nanostructures within the composites. The insets in Figure 2a,b display images of Au and Ag single NPs within the composites (top panels) along with the spatial distribution of the LSPR signal (bottom panels). Highly localized plasmon resonances become apparent at the external surfaces of the NPs (centered at 2.35 and 2.90 eV for Au and Ag, respectively), expanding around within the acrylic matrix a few nanometers for both types of materials. The observed distribution around the NPs correlates with a strong dipole contribution for both materials. Spectral differences between UV–vis and EELS arise from the far-field nature of UV–vis in contrast to the near-field nature of EELS. Since the maximum electric field intensity depends differently on the particle polarizability, the electric field may be maximized at different wavelengths upon photon and electron excitation.^53^ Different authors attribute the shift of near-field-related signals (such as EELS) to radiation-damping effects explained through the harmonic oscillators model^54^ or to evanescence waves dominating the interactions (instead, far-field measurements rely on propagating waves).^55^ The dominance of dipolar and quadrupolar modes with the opposite phase in the near-field but the same phase in the far-field may also explain the spectral shift between the far- and near-fields.^46^ Due to these discrepancies between far- and near-field performance, the most convenient technique to address the plasmonic response of a material may depend on its sought application and whether its functional principle takes advantage from far- or near-field phenomena. Therefore, near-field measurements become particularly beneficial for the smart design of materials whose performance is based on the near-field enhancement related to the LSPRs, such as systems intended for optical trapping^10^ or SERS detection since the analyte excitation is achieved at the LSP energy driving the SERS effect.^16^ Actually, the optimal performance of SERS nanoantennas depends not only on the exact resonant frequency but also on the near-field intensity.^56^ On the other hand, the far-field measurements become particularly relevant for the characterization of materials intended for applications such as photocatalysis, energy harvesting, or metasurfaces,^57^ since their response is governed by the light propagation within the material.

### Influence of the Metal, NP Diameter, Actual Environment, and Detection Technique on the Plasmonic Response

**Figure 3:**
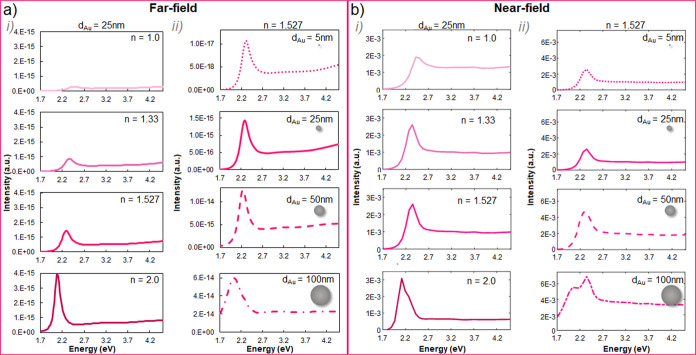
Simulated (a) far-field and (b) near-field LSPR spectral evolution (i) for 25 nm diameter Au NPs embedded in media with *n* = 1.0, 1.33, 1.527, and 2.0 (from top to bottom) and (ii) for 5, 25, 35, and 100 nm Au NPs (from top to bottom) embedded in a medium with 1.527 refractive index.

**Figure 4:**
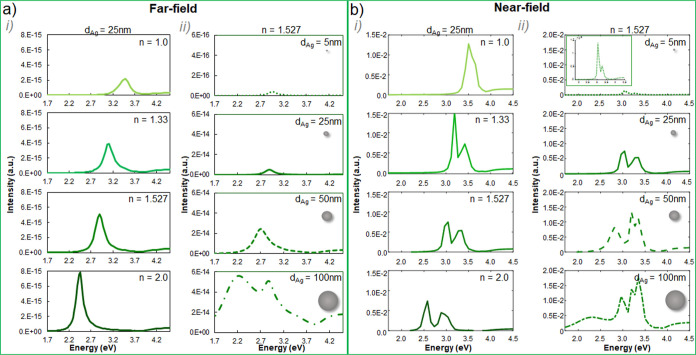
Simulated (a) far-field and (b) near-field LSPR spectral evolution (i) for 25 nm diameter Ag NPs embedded in media with *n* = 1.0, 1.33, 1.527, and 2.0 (from top to bottom) and (ii) for 5, 25, 35, and 100 nm Ag NPs (from top to bottom) embedded in a medium with 1.527 refractive index.

The plasmonic response of differently shaped Ag and Au NPs has been extensively studied during the past years, and the systems are well-described facing colloidal dispersions or pure NPs (i.e., in air or vacuum), while most reported studies on the role of the environment focus on substrate-supported systems. In order to get a deeper understanding of the plasmonic response of spherical Au and Ag NPs within polymer composites, we compare the calculated far- and near-field spectral lines for a variety of systems containing either Au (Figure 3) or Ag NPs (Figure 4) in different media. Importantly, most polymers possess refractive indexes within the range of 1.3–1.6 while few polymers show higher refractive indexes, usually between 1.7 and 1.8^58^ (see Table S1 Supporting Information). The usefulness of high refractive index polymers for their integration in advanced optoelectronic devices has pushed the design of novel polymer systems with refractive indexes close to 2^59^ (i.e., *n* = 1.936,^60^*n* = 1.98^61^). Therefore, we have extended our calculations to cover materials with refractive indexes between 1 and 2, by considering the particular cases of air (*n* = 1), water, and low refractive index polymers (*n* = 1.33), the acrylic resin experimentally used (*n* = 1.527), and *n* = 2. We have considered two separated cases of study: (i) 25 nm diameter NPs at different refractive indexes media (namely, *n* = 1, 1.33, 1.527, and 2) and (ii) a material matrix with *n* = 1.527 containing different diameter NPs ranging between 5 and 100 nm (*d* = 5, 25, 50, and 100 nm).

Figure 3i displays the extinction UV–vis (far-field, a) and EELS (near-field, b) spectral response expected for 25 nm Au NPs at media with 1, 1.33, 1.527, and 2 refractive indexes (from top to bottom). There is an energy red shift of the LSPR noticeable at both, the far- and near-field, for increasing refractive indexes, while the signal intensity increases. The maximum resonant energies obtained are listed in Table 1. The LSPR energy of 25 nm Au NPs red-shifts 0.37 eV in the far-field and 0.30 eV in the near-field by changing the surrounding refractive index from 1 to 2. No significant discrepancies between the far-field and near-field resonant energy (|Δ*E*| < 0.06 eV) were found for this diameter regardless of the refractive index of the medium.

**Table 1:** Far-Field (FF *E*_max_) and Near-Field (NF *E*_max_) LSPR Energy for Au NPs with Diameters Ranging between 5 and 100 nm, Placed at Different Media (*n* = 1–2), along with the Energy Shift between FF and NF Signals, Δ*E*

*d*/nm	*n*	FF *E*_max_/eV	NF *E*_max_/eV	|Δ*E*|/eV
25	1.0	2.45	2.42	0.03
25	1.33	2.37	2.35	0.02
25	1.527	2.29	2.35	0.06
25	2.0	2.08	2.12	0.04
5	1.527	2.31	2.35	0.04
50	1.527	2.24	2.25	0.03
100	1.527	2.04	2.04	0.00

Figure 3a(ii) shows the calculated far-field and near-field spectra from Au NPs with diameters ranging from 5 to 100 nm (namely, 5, 25, 50, and 100 nm) embedded within the same matrix, with *n* = 1.527, in order to evaluate possible LSPR size effects at the nanocomposites. As expected, the LSPR energy from Au NPs red-shifts with increasing diameters (see Table 1) due to retardation effects, and the signal becomes stronger (i.e., more intense) as well as broader. The LSPR energy shifts 0.27 eV at the far-field if comparing 5 nm diameter Au NPs (LSPR energy centered at 2.31 eV) with 100 nm NPs (2.04 eV), and the near-field energy shift reaches 0.31 eV. Interestingly, the resonant energy for Au NPs with diameters ranging between 5 and 100 nm in air (*n* = 1) is not expected to vary with the NP size, being centered at 2.45 and 2.42 eV at the far-field and near-field, respectively, according to our calculations (more details are provided in the following). Remarkably, LSPR retardation effects in Au NPs may already be expected for NPs with diameters ranging between 5 and 100 nm in higher refractive index media.

Overall, the LSPR from small Au NPs (i.e., *d* < 50 nm) remain almost unaltered with the size and refractive index apart from the slight red shift and increased peak intensity due to retardation effects.^23^ The retardation effects are stronger for larger NPs since the oscillation frequency becomes comparable to the time needed for the dipole excitation. Indeed, Au NPs large enough may show contribution from higher order polar modes, as well as higher damping rates (radiative damping).^52^

Moving to the Ag nanocomposites, we have computed far-field and near-field spectral signals from Ag NPs, summarized in Figure 4a,b, respectively. Once again, we have considered two cases of study, (i) involving Ag NPs with constant diameter (25 nm) at different refractive indexes media (namely, *n* = 1, 1.33, 1.527 and 2) and (ii) varying diameter NPs between 5 and 100 nm (specifically, 5, 25, 50, and 100 nm diameters) embedded within acrylic resin (*n* = 1.527). The LSPR from 25 nm Ag NPs red-shifts with increasing *n* (Figure 4i) up to 1 eV at the far-field (0.9 eV at the near-field) within the studied range. The signal at the far-field becomes more intense for higher *n* due to the increased NP polarizability for higher refractive indexes.^62^ Interestingly, the spectral response of 25 nm Ag NPs at the near-field is strongly affected by *n*, and the signal evolves from one single peak centered at 3.50 eV (*n* = 1) toward two separated peaks, centered at 2.58 and 2.88 eV (*n* = 2), suggesting the contribution of multipole order modes^51^ whose excitation is favored at higher refractive indexes media. It is known that metallic NPs large enough show dipolar and quadrupolar resonant modes due to retardation effects: as the NP size increases, the electric field is no longer homogeneously distributed within the NP, resulting in phase retardation which red-shifts and broadens the dipolar resonance and promotes the appearance of higher order resonant modes.^63^ The elementary intrinsic properties of a given conductive nanosphere embedded in a certain dielectric material dictate the requested diameter for a resonant mode to be expected.^64^ Consequently, the threshold diameter for achieving higher order mode excitation is dependent on the material composing the active plasmonic nanostructure itself. Moreover, the higher the refractive index of the medium embedding the NPs, the larger the expected energy discrepancies between different order resonant modes (i.e., larger differences are expected between the resonance energies of dipolar and quadrupolar modes as the refractive index of the medium increases).^64,65^ Therefore, while placing the electron probe close to the surface of Ag NPs in air (*n* = 1) results in LSPRs dominated by dipole modes and, thus, single spectral peaks; increasing the refractive index of the media promotes the contribution of higher order modes^65^ and the signal damping.^23,51^ This LSPR splitting into several modes at the near-field may drive prominent differences between the far- and near-field responses of AgNPs material systems, which is remarkable for higher refractive index media and larger NPs. In contrast, AuNPs of the same size (i.e., 25 nm diameter) only show dipolar resonances (see Figure 3) regardless of the refractive index of the medium within the tested range (1 < *n* < 2). However, larger AuNPs may also show quadrupolar resonances, particularly if they are placed at high refractive index media (for example, 100 nm diameter AuNPs at a medium with refractive index 2, see Figure S5 at the Supporting Information). In fact, according to the literature, AuNPs in water (*n* = 1.33) must exceed 100 nm to show dipolar and quadrupolar spectral contributions,^66^ whereas quadrupolar contribution might be expected for AgNPs larger than 30 nm diameter in the same medium (water, n = 1.33).^67,68^ Importantly, both active modes separately may be suitable, for instance, to achieve fluorescent enhancements at selected wavelengths.^69^ More details on the differentiated modes contributing to the AG LSPR signal can be found in the Supporting Information

LSPR size effects for Ag NPs located within a medium with *n* = 1.527 are addressed in Figure 4ii. Narrower and dimmer resonances are estimated for smaller NPs (5 nm diameter), while the signals become strongly broad for the largest NPs considered (100 nm). The expected energy red shift for larger NPs^70^ is clearly noticeable at the far-field, reaching a 0.79 eV signal red shift compared 5 nm (resonant energy centered at 2.98 eV) to 100 nm NPs (2.19 eV). Aside from the signal red shift for increasing NP sizes, larger Ag NPs result in more complex spectral shapes (i.e., multiple peaks). For instance, two differentiated modes (centered at 3.04 and 3.35 eV; see Table 2) are distinguished at the EELS signal (near-field) from 25 nm Ag NPs in a medium with *n* = 1.527. Importantly, far-field measurements access mainly dipolar excitations, whereas near-field techniques measure higher order modes.^71^ Accordingly, the calculated far-field spectrum for this system shows a single peak centered at 2.9 eV (i.e., dipole mode). For the 100 nm diameter Ag NPs, both the far- and the near-field spectra show signatures from dipole (broader and at lower resonant energy) and quadrupole (sharper and at higher resonant energy) resonances, whose relative contribution is opposite at the far-field (dominated by the broad dipolar mode centered at 2.19 eV) and near-field (governed by the higher energy peak at 2.96 eV) (Figure 4ii). Consequently, the LSPR energy difference between 5 and 100 nm Ag NPs placed in a 1.527 refractive index environment is 0.31 eV at the near-field, in contrast to the 0.79 eV red-shift expected at the far-field.

**Table 2:** Far-Field (FF *E*_max_) and Near-Field (NF *E*_max_) LSPR Energy for Ag NPs with Diameters Ranging between 5 and 100 nm, Placed at Different Media (*n* = 1–2), along with the Energy Shift between FF and NF Signals, Δ*E*^a^

*d*/nm	*n*	FF *E*_max_/eV	NF *E*_max_/eV	|Δ*E*|/eV
25	1.0	3.48	3.50	0.02
25	1.33	3.12	3.19	0.07
25	1.527	2.90	3.04	0.14
25	2.0	2.45	2.58	0.13
5	1.527	2.98	3.04	0.06
50	1.527	2.71	3.19	0.48
100	1.527	2.19	3.35	1.16

a Note that in case of mode splitting, the values included refer to the most intense contribution

Therefore, Ag spectra show prominent changes depending on the NP diameter over the full range of sizes studied and the refractive index of the media, and plain differences may be expected for the plasmonic response of these systems depending on the detection technique (i.e., whether it relies on far-field or near-field measurements).

**Figure 5:**
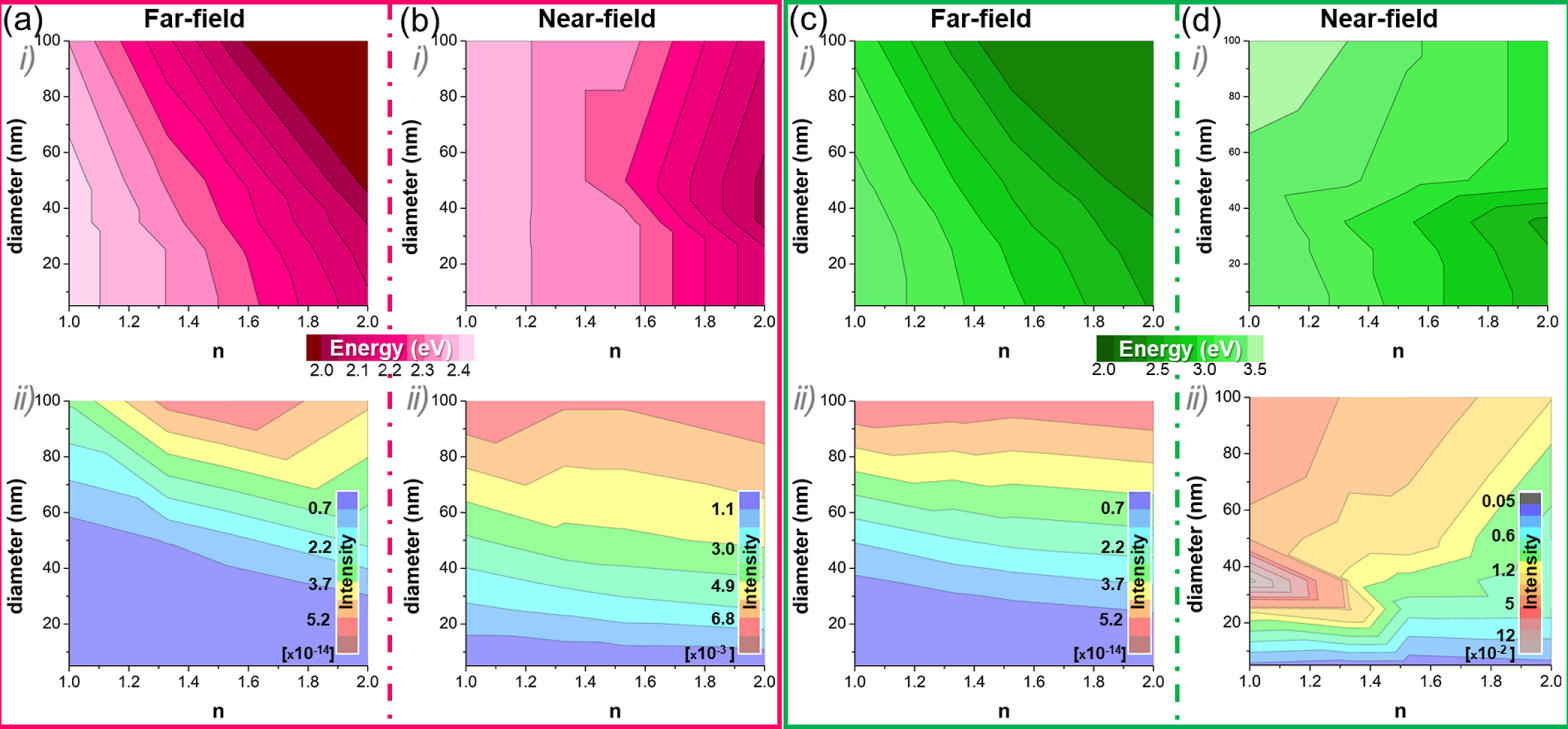
LSPR energy (i) dependence as a function of the NP diameter (ranging between 5 and 100 nm) and *n* of the surrounding media (1 < *n* < 2) for Au (a, b) and Ag (c, d) at the far-field (a, c) and near-field (b, d). Panels (ii) display the color maps signal intensity (color codes at the insets).

To better explore the possibilities rendered by Au and Ag plasmonic systems and the experimental capabilities of UV–vis absorption and EELS techniques, we spread the comparison of both computed signals (far-field UV–vis extinction spectra and near-field EELS spectra) over all the possible materials systems resulting from the 4 different environments (i.e., NPs placed at media with refractive index 1, 1.33, 1.527, and 2) and over different NP diameters, namely, 5, 25, 35, 50, and 100 nm. Figure 5 displays the far- (a, c) and near-field (b, d) LSPR energy of Au (a, b) and Ag (c, d) NPs as a function of both the refractive index of the surrounding media and the NP diameter, including the peak center expressed in energy units (i) and its intensity (ii), displayed as rainbow-color map. Ag plasmon-related signal at the near-field is stronger (higher intensity) than Au in all cases considered, owe to the superior Ag intrinsic dielectric properties.^72^

The resonant red shift at fixed NP diameters (5, 25, 50, and 100 nm) for increasing refractive indexes becomes evident for both material systems. Moreover, larger shifts with *n* are expected for larger NPs since retardation effects become more relevant. It is worth noting the presence of two differentiated contributions to the signal for the 100 nm Au NPs at *n* = 2, with a larger peak at 1.70 eV and a smaller one at 2.08 eV, likely arising from dipolar (at 1.70 eV) and quadrupolar (at 2.08 eV) resonant modes^52^ (see Supporting Information). In this case, the dominant contribution to the signal arises from the lower energy mode at 1.70 eV (i.e., dipole) at the far- and near-field. As already mentioned, the contribution of different resonant modes is favored for Ag, becoming more acute as the refraction index of the medium increases, in agreement with previous reports.^73^ Note that the displayed results account for the most intense LSPR if several modes are excited (see Supporting Information for further details on the other modes). The data evidence the differentiated behavior of Ag NPs at the far- and near-field arising from the prevalence of dipolar contribution at the far-field, in contrast to the enhanced role of higher order modes at the near-field.

Focusing on fixed *n* values (namely 1.0, 1.33, 1.527 and 2.0) allows for the evaluation of size effects on different media, probing larger red shifts with the NP size at higher refractive index environments. The same trends are observed at the far- and near-fields.

Based on the systematic comparison established involving far- and near-field signals, we can address a few differences between both materials systems. Subtler differences are expected on the LSPR from Au NPs upon varying the NP diameter (between 5 and 100 nm) and refractive index (within 1–2) of the medium hosting the NPs, regardless of the measuring technique (UV–vis or EELS). On the contrary, Ag spectra show prominent changes depending on the diameter of NPs over the full range of sizes studied and the refractive index of the media. Increasing the refractive index favors the resonance of higher order modes (i.e., quadrupole), and the effect is stronger for larger NPs, particularly at the near-field. Indeed, the LSPR energy from Ag NPs is highly dependent on the refractive index of the media, and even 25 nm diameter NPs red-shift about 0.1 eV when placed within a medium with *n* = 2 as compared to *n* = 1, while the estimated red shift for Au counterparts is 0.01 eV. As already mentioned, the shift is also size dependent, meaning that NPs with different sizes would experience different dependence on the refractive index of the medium, particularly when dealing with Ag.

Discrepancies between far- and near-field measurements have been previously explained attending to the contribution of the real and imaginary part of the polarizability to the signal since the real part of the polarizability dominates near-field signals while the imaginary part does for the far-field.^53^ Accordingly, we have found larger differences between UV–vis and EELS signals from Ag NPs than those from Au NPs. Au NPs show LSPRs centered about 1.7 eV (at the far-and near-field) for 100 nm NPs within a medium posing a refractive index of 2 and at approximately 2.4 eV for 5 nm NPs in air (*n* = 1.0). Ag LSPRs lie within 2.4/2.5–3.5 eV at the far-/near-field for the tested conditions, going from 100 nm diameter NPs in a 2.0 refractive index media (which show complex spectral shapes) to 5 nm NPs in air (*n* = 1). Simulated far- and near-field spectra for the smaller NPs at lower refractive indexes (i.e., 5 nm NP at *n* = 1) and for the larger diameter at higher refractive indexes considered (i.e., 100 nm NP at *n* = 2), for Au and Ag, are provided in the Supporting Information.

Thus, the spectral shape from Ag NPs accounts for the contribution of multiple order resonant modes (i.e., dipole, quadropole) to the LSPR, whose relative contribution to the overall LSPR is strongly size dependent.^71^ Moreover, the modes are better resolved when increasing *n*. This imposes the advantage of spreading the attainable operation range of the designed systems if achieving narrow size distributions and environmental homogeneity (otherwise the plasmonic response will be easily broadened). Nonetheless, wider resonances might be useful for broadband filtering. In contrast, Au NPs are less sensitive to size effects and, indeed, no meaningful differences are expected on the LSPR from 25–50 nm diameter Au NPs; neither measured at the far-field (UV–vis), nor at the near-field (EELS).

Shaping the nanocomposites at will since they are provided with 3D printing capabilities spreads their application range if made from materials that fulfill the functional requirements for the sought performance. A deeper knowledge of the phenomena driving their physical properties increases the attainable degree of control over the functionality. Within this context, a better understanding of the spectral properties of Au and Ag polymer nanocomposites envisages future advances on the design of on-demand functional materials suitable for 3D printing. As an example, 3D-printing-suitable combinations of materials enables achieving selective wavelength filtering, appealing, for instance, for the development of colorblindness correction lenses.^7^ While the study involves the use of organic dyes to achieve the desired optical behavior, other reported studies show the implementation of Au and Ag nanocomposites with similar purposes,^74−76^ probing the suitability of these materials working as selective optical filters, whose optimization and customization will be better achieved, accounting for our results.

To summarize, plasmonic materials based on Au NPs allow for broader NP size distributions, keeping narrow LSPRs, while the resonant energy can be tuned to some extent by changing the refractive index of the surrounding media. Ag-based plasmonic nanocomposites require higher control over the composite homogeneity if narrow resonances are desired, while they allow for tuning the LSPR between 2.4 and 3.5 eV. It may be noted that larger Ag NPs than 35 nm (diameter) within the acrylic resin may reach lower resonant energies for the dipole mode while promoting the resonance of higher order modes. Remarkably, the quality factor of the resonances, *Q*, defined as the resonant energy over the spectral line width, imposes a figure of merit for certain applications such as SERS, claimed to be proportional to *Q*^4^.^77^ The design of materials reaching spectral resolved resonant modes or obtaining single dipole resonances allows improvement of the quality factors for such applications. In our particular case, the size of the NPs might be optimized by suitable post-treatment steps or by fine-tuning the printing procedures favoring the growth of larger NPs. Moreover, the autocatalytic growth of the NPs can be also greatly controlled by immersing the composites in suitable salt baths, imposing another route for tailoring the NP size.^78^ As already mentioned, larger NPs favor the light scattering, enhancing far-field sensing. Alternatively, the development of nanocomposites containing different fillers such as Ag_*x*_Au_*y*_ alloy NPs should provide resonances within the frequency gap between Au and Ag LSPRs.^79^ This is the case of Teflon films containing Ag_*x*_Au_*y*_ NPs, whose UV–vis response has been reported to range between 3.1 and 1.9 eV for increasing Au content,^28^ and showing wider resonances for increasing alloying.

## Conclusions

We address the plasmonic response of Au- and Ag-polymer nanocomposites suitable for 3D printing with active LSPR at 2.3 and 3.0 eV, respectively. We compare UV–vis (far-field) and EELS (near-field) experiments and simulations. The plasmonic properties of Au nanocomposites are properly addressed by UV–vis and EELS. However, the absorbance of the acrylic resin dampens the LSPR related to Ag NPs, hindering the accurate UV–vis measurement of Ag nanocomposites. Nonetheless, we measured the LSPR from single NPs within the acrylic resin for both nanocomposites by means of EELS, providing 2D maps of the LSPR distribution around the NPs. We carefully investigated size effects on the combinations of materials explored, evidencing the higher sensitivity of Ag LSPRs to extrinsic factors compared to Au. Consequently, Au-polymer nanocomposites may allow wider size distribution of NPs preserving narrow resonances, while Ag-polymer nanocomposites permit easier selective tuning over the resonant energy by changing the NP diameter.

The comparison established also evidences the differentiated near- and far-field response of each material system. We showed that facing Ag-acrylic resin nanocomposites, the optical activity of the matrix interferes with the LSPR from Ag NPs. Moreover, discrepancies between UV–vis and EEL spectra highlight the relevance of properly addressing the plasmonic properties based on far- or near-field measurements, depending on the intended applications for the material under study (i.e., whether the working principle relies mainly on light scattering, far-field, such as solar cells, or on the electromagnetic near-field enhancement, as is the case for SERS detectors).

## Experimental Section

### Materials

#### Synthesis of Au and Ag Nanocomposites

Nanocomposites were synthesized by dissolving either 0.1 wt % KAuCl_4_ (Acros) or 3 wt % AgClO_4_ (Alfa Aesar) in a commercial acrylic resin (clear photopolymer standard resin, XYZprinting). The mixtures were sonicated for 30 min in an Ultrasonic Cleaner USC500T provided by VWR, working at 45 kHz. Solid specimens for EELS and UV–vis measurements were printed by SL using a Nobel 1.0 (XYZprinting), equipped with a 405 nm laser with an output power of 100 mW and a spot size that allows for an XY resolution of 300 μm. All of the samples were printed with a layer height of 100 μm. Once printed, the samples were washed in isopropanol (Scharlab) for several minutes. A postprocessing of the samples was performed for 60 min at 60 °C inside a UV chamber (FormCure, Formlabs) with a light source of 405 nm and a power of 1.25 mW/cm^2^. Alternatively, the Au or Ag resin precursor was poured into a transparent polypropylene mold (8 mm diameter, 3 mm height) and cured inside the UV chamber for 60 min at room temperature, obtaining the same nanocomposite as in SL. In all cases, the Au nanocomposites underwent a thermal treatment at 180 °C for 1 h to reduce Au^3+^ into AuNPs, as previously reported.^47,48^

### Characterization Methods

#### UV–Vis Measurements

Spectra were measured by using a Varian Cary 50 Conc spectrophotometer. The range between 200 and 800 nm was monitored with a scan rate of 10 nm/s.

#### STEM–EELS Experiments

Electron transparent specimens from Au- and Ag-acrylic resin samples were cut by ultramicrotomy, known to provide thin uniform sections from polymer-based materials.^80^ Low-loss electron energy-loss spectroscopy (LL-ELLS) measurements were carried out on a 60–300 kV Titan Cube FEI transmission electron microscope operated in scanning mode (Scanning Transmission Electron Microscopy, STEM) at 200 kV, equipped with a probe corrector and a Gatan Continuum energy filter (i.e., DualEELS Spectrometer). The equipment was set up to achieve convergence and collection semiangles of 20.5 and 41 mrad, respectively. The exposure time and beam current were optimized for each sample, being on the order of milliseconds and tens of picoamperes, respectively, and two energy dispersions were used, 0.005 and 0.01 eV/ch. The spectral resolution extracted from the full-width at half-maximum (fwhm) of the zero-loss peak (ZL) was 0.7 eV, while keeping a subnanometer spatial resolution. The experiments were carried out by 2D spectrum imaging, meaning that the electron probe is scanned over the region of interest, recording one spectrum per pixel. Annular dark field (ADF) images were simultaneously acquired. Extracted spectra from the region of interest (namely, the external surface of the NPs) provide the experimental near-field measurements considered.

#### Data Treatment

Data were processed by using Gatan Digital Micrograph software. In order to increase the spectral signal-to-noise ratio, the data have been denoised by applying principal component analysis (PCA), implemented with the Digital Micrograph software package. After aligning the spectrum image stacks according to the ZL position, spectral signals were extracted by removing the background, fitting a power-law function to the ZL tail.

## Calculations

Far- and near-field spectra were computed accounting for spherical NPs with diameters ranging between 5 and 100 nm (namely, 5, 25, 35, 50, and 100 nm diameters), made from Au and Ag and placed at media with 1.0, 1.33, 1.527, and 2.0 refractive indexes, (*n*). The extinction cross-section UV–vis spectra have been calculated following Mie’s formalism,^81,82^ which relates to the far-field response of the Au- and Ag-nanocomposites. EELS signals have been calculated as the loss probability of the electrons (accelerated at 200 kV) crossing the material by means of boundary element method (BEM)^83^ implemented at the MNPBEM Matlab toolbox,^84,85^ rendering near-field information from individual NPs at the different environments. The metal dielectric constants of both, Au and Ag, were taken from the toolbox, and they may be found in the Supporting Information along with further details on the EELS simulations. The distance from the electron probe to the center of the NPs, so-called impact parameter (*b*), was set to lie close to the NP-polymer interface (i.e., at the external surface of the NPs); while the effect of the impact parameter on the resonant energy was further checked for 25 nm diameter NPs at different refractive index media (*n* = 1, 1.527, 2.0), shown in the Supporting Information. While we checked the suitability of the quasi static approximation for computing the response of the smaller NPs (i.e., diameters shorter than 25 nm), we performed all calculations including retardation effects in order to evaluate, in deeper detail, the spectral shape.

## Supporting Information Available

Au and Ag dielectric constants used for the calculations, evaluation of the impact factor considering different media (i.e., 1 < *n* < 2), EELS simulations by means of the quasi-static approximation as compared to the full resolution of Maxwell equations, further EEL spectral features of Ag NPs (for increasing *n*), and compared far- and near-field spectra of 5 and 100 nm diameter Au and Ag NPs placed at both, *n* 1 and 2 (PDF)
