# Supplementary material for: Plasmonic Characterization of 3D Printable Metal–Polymer Nanocomposites
Source: ACS Mater Au. 2024 May 15;4(4):424–35. doi: 10.1021/acsmaterialsau.4c00007 (PMC11240405; doi:10.1021/acsmaterialsau.4c00007)
Supplement: Supplementary file 1 — mg4c00007_si_001.pdf [file mg4c00007_si_001.pdf]

# Supporting Information: Plasmonic Characterization of 3D Printable Metal-Polymer Nanocomposites

María de la Mata[1]*, Alberto Sanz de León[1], Luisa M. Valencia-Liñán[1], Sergio I. Molina[1]

[1]Departamento de Ciencia de los Materiales, I. M. y Q. I., IMEYMAT, Universidad de Cádiz, Campus Rio San Pedro, 11510 Puerto Real, Spain.

*Corresponding author: maria.delamata@uca.es

## Contents

**Optical properties of some polymers** **2**

Examples of different polymers with refractive index values ranging between 1.3 and 2. . . . . . 2

**Details of the EELS simulations** **2**

Dielectric constants . . . . . . . . . . . . . . . . . . . . . . . . . . . . . . . . . . . . 3

Au (a) and Ag (b) dielectric constants used for the BEM calculations. (S. 1) . . . . . . . 3

Impact factor . . . . . . . . . . . . . . . . . . . . . . . . . . . . . . . . . . . . . . . 4

LSPR as function of *b* for different refractive indexes (S. 2) . . . . . . . . . . . . . . . . . . 4

Retardation effects . . . . . . . . . . . . . . . . . . . . . . . . . . . . . . . . . . . . 5

Quasi static approximation vs full resolution of Maxwell equations (S. 3) . . . . . . . . . . 5

**Other EELS simulations** **6**

EEL spectral features of Ag NPs . . . . . . . . . . . . . . . . . . . . . . . . . . . . . . 6

Spectral shape evolution of Ag NPs for increasing refractive indexes (S. 4) . . . . . . . . . 6

Small NP at low refractive index medium *vs* large NP at high refractive index medium . . . . . 7

Au NP (S. 5) . . . . . . . . . . . . . . . . . . . . . . . . . . . . . . . . . . . . . . . 7

Ag NP (S. 6) . . . . . . . . . . . . . . . . . . . . . . . . . . . . . . . . . . . . . . . 8

# Optical properties of some polymers

## Examples of different polymers with refractive index values ranging between 1.3 and 2.

**Table 1:** Refractive indexes of different polymers[1, 2, 3, 4, 5].

| Polymer | n |
|---:|:---:|
| Fluorinate | 1.31-1.4 |
| PLA | 1.35-1.45 |
| Epoxy | 1.45-1.6 |
| Acrylic | 1.47-1.57 |
| PP | 1.5-1.51 |
| PE | 1.51-1.54 |
| Polyamide | 1.52-1.53 |
| PVC | 1.53-1.55 |
| ABS | 1.54 |
| PS | 1.55-1.59 |
| PET | 1.55-1.64 |
| PC | 1.58-1.6 |
| PEEK | 1.65-1.77 |
| Poly(Arylene sulfide)s | 1.66-1.72 |
| X-polymethacrylates | 1.68-1.77 |
| High refractive index hybrids | 1.94, 1.98 |

# Details of the EELS simulations

The EEL spectra have been simulated by means of boundary element method (BEM) approach, implemented at the MNPBEM toolbox [6, 7]. The electron energy loss probability is calculated either by solving full Maxwell's equations or its quasi static limit at the boundary of the nanostructures discretized by boundary elements (particularly, we employed 256 for sphere triangulation). The material system is defined by the morphology of the nanostructure (i.e., shape and size) and the optical properties of both materials composing the system (i.e., the dielectric constant of the metal nanostructure and the refractive index of the surrounding medium, $n$). Other required input parameters are the electron velocity of the scanning probe (directly related to accelerating voltage, 200 kV in our study), as well as the impact parameter (the distance between the electron probe and the nanostructure).

## Dielectric constants

Metal dielectric constants employed for the calculations provided by the MNPBEM toolbox [6, 7].

### Au (a) and Ag (b) dielectric constants used for the BEM calculations. (S. 1)

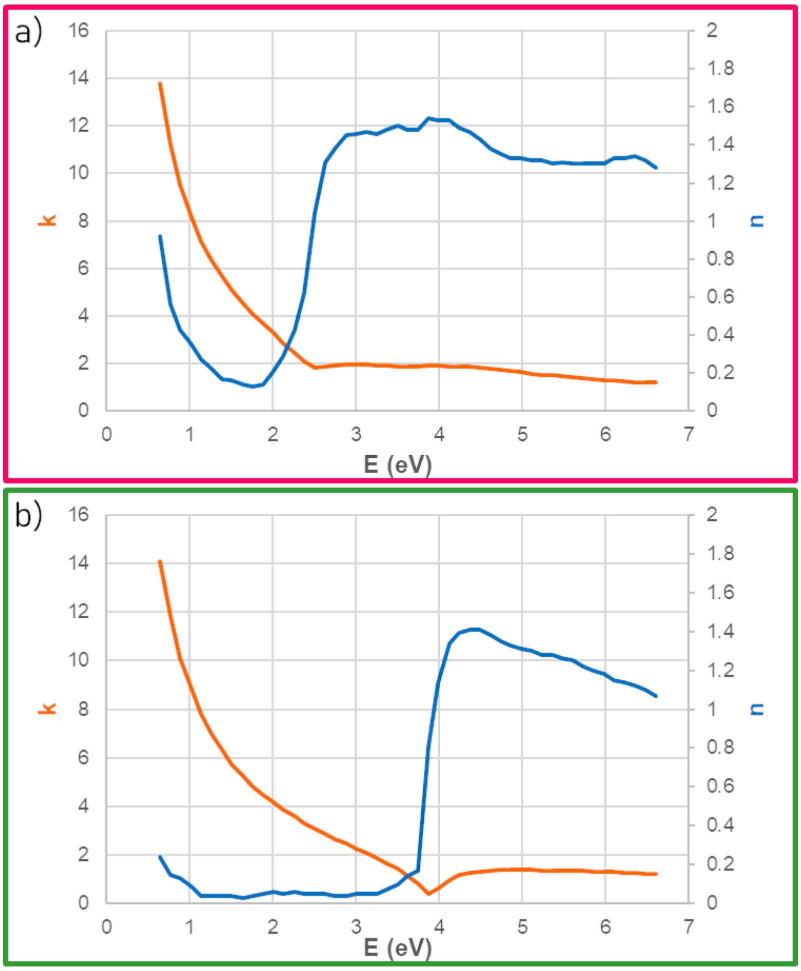


**S. 1:** Au (a) and Ag (b) dielectric constants used for the BEM calculations.

## Impact factor

It is well-known that the obtained EELS spectra is strongly dependent on the point of impact of the electron beam. Therefore, we have evaluated the electron loss probability as function of the distance between the electron beam and the NP (impact factor) for 25 nm diameter Au (**Fig.**2a)) and Ag (**Fig.**2b)) NPs within different refractive index media (namely, 1.0, 1.527 and 2.0), using the quasi static approximation (good enough for 25 nm diameter NPs, as shown at Supp. Info. Fig. 3).

**LSPR as function of *b* for different refractive indexes (S. 2)**

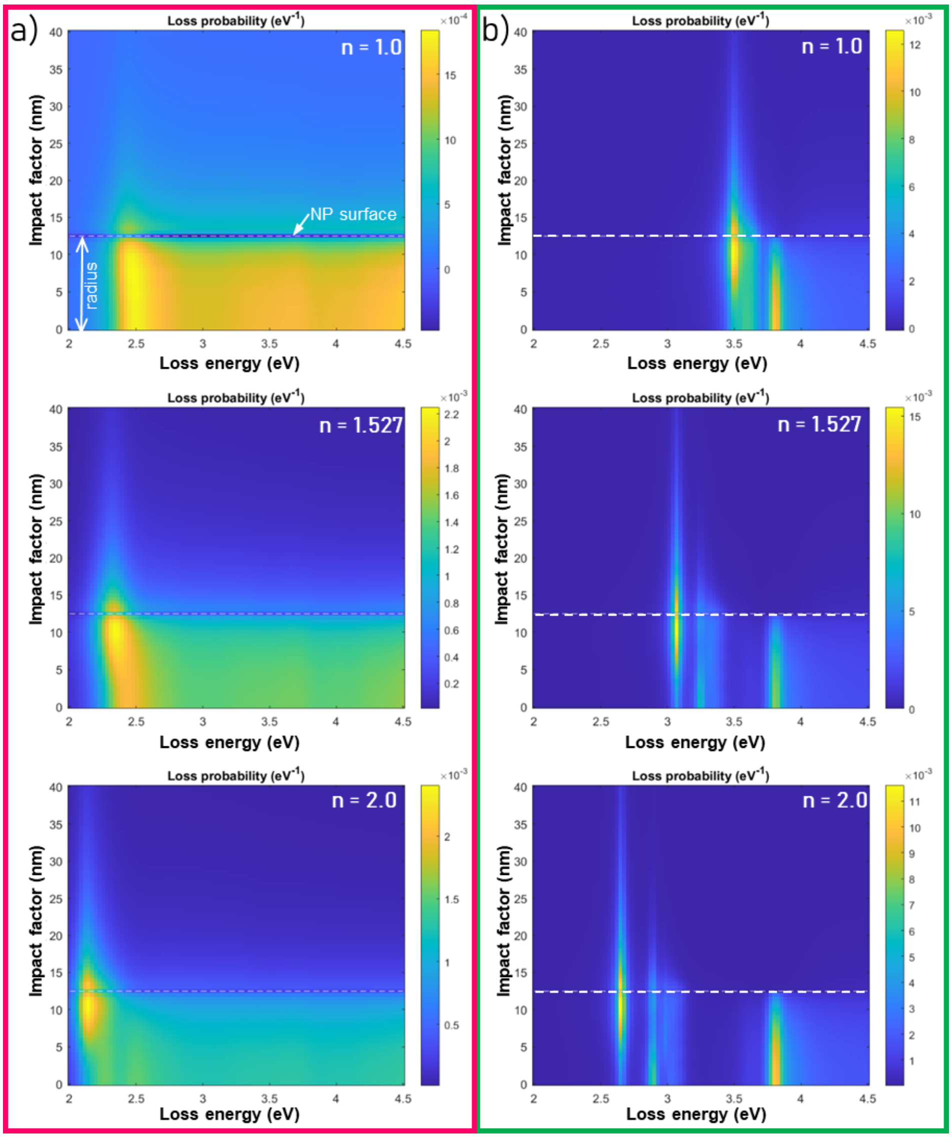


**S. 2:** Energy loss and its probability for a single 25 nm diameter Au (a) and Ag (b) NP in different media ($n$ = 1, 1.527 and 2, from top to bottom) as function of the impact parameter (i.e., distance between the electron beam and the NP). Larger refractive indexes red-shift the LSPR energy and may induce the peak splitting.

## Retardation effects

### Quasi static approximation vs full resolution of Maxwell equations (S. 3)

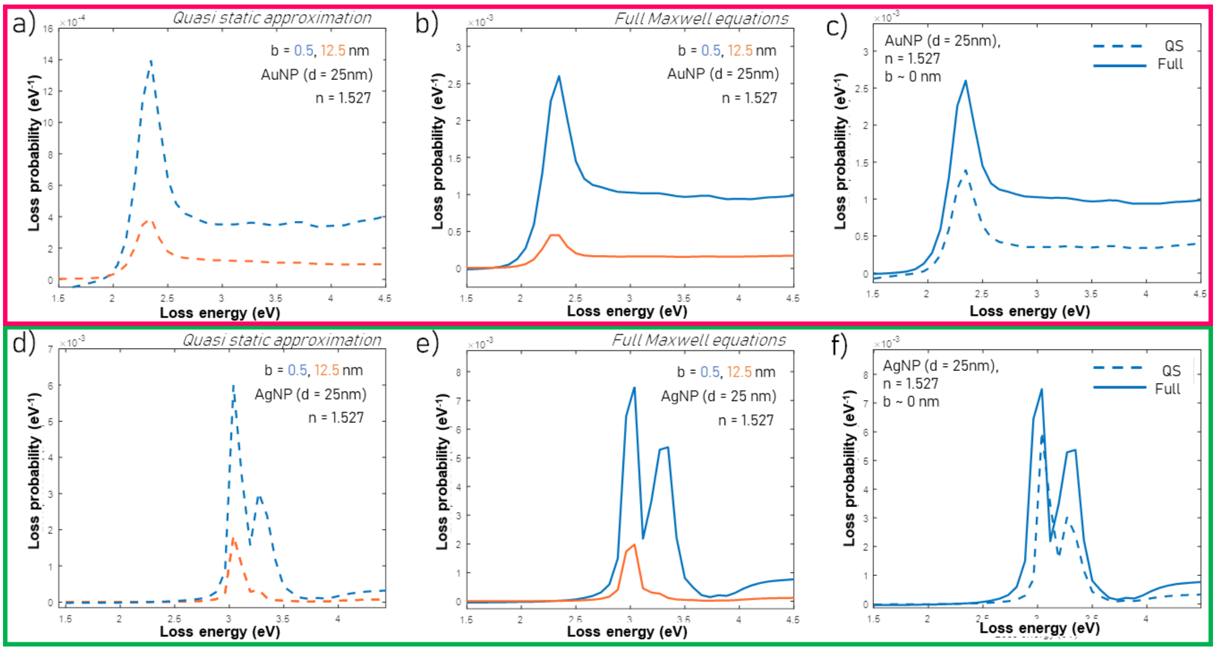


**S. 3:** Simulated EEL spectra for 25nm diameter Au (a-c) and Ag (d-f) single NPs embedded in a medium with 1.527 refractive index. The spectra have been calculated by means of the quasi static approximation (a, d) and by solving full Maxwell equations (b, e), for two impact factors in each case (i.e., at the surface of the NP and 12.5 nm apart from the surface, plot in blue and orange, respectively). The spectra at the NP surface calculated by both approaches are displayed at c) and f) for ease comparison. The quasi static approximation is good enough for the diameters considered (for larger diameters the quasi static approach may fail, particularly for the Ag).

# Other EELS simulations

## EEL spectral features of Ag NPs

### Spectral shape evolution of Ag NPs for increasing refractive indexes (S. 4)

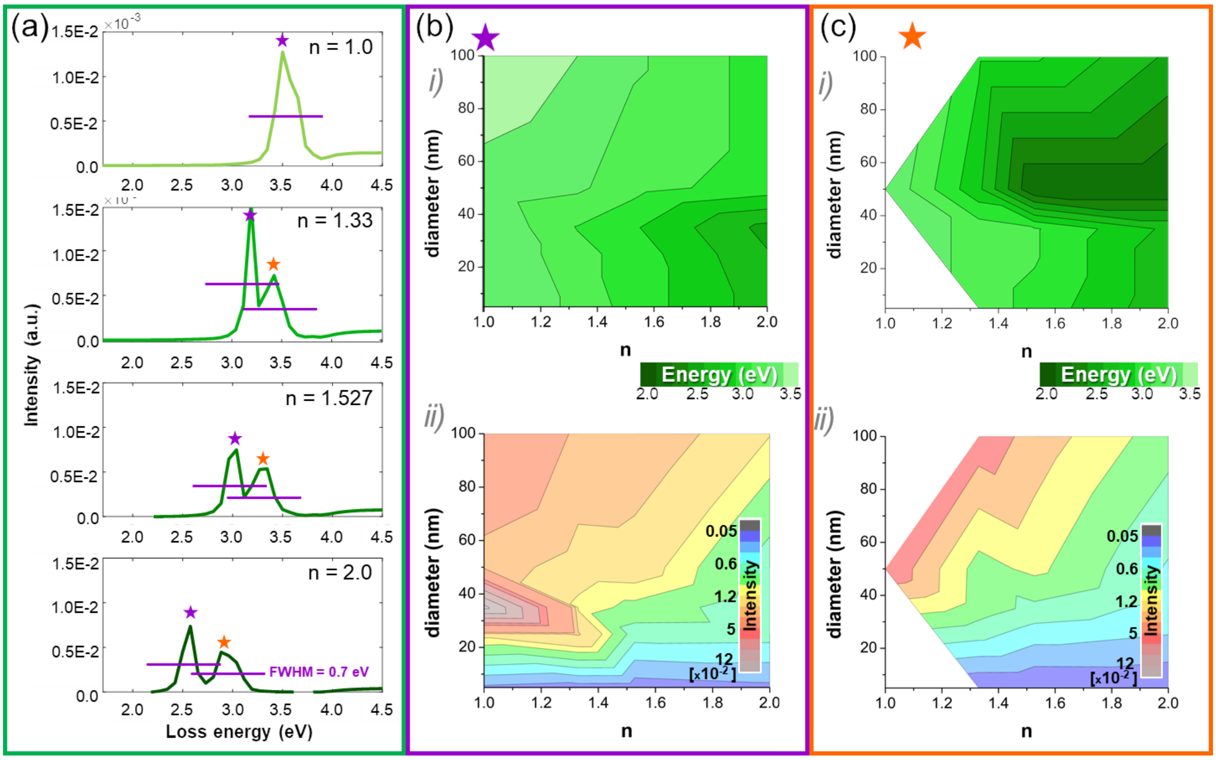


**S. 4:** a) Simulated EELS spectra for a Ag NP (d=25nm) in different media (n = 1, 1.33, 1.527 and 2, from top to bottom, respectively), evidencing the splitting of the LSPR into 2 separated peaks 0.3 eV apart, indicated with purple and orange stars. The convolution of both contributions might lead to broad experimental peaks. b) and c) displayed the estimated LSPR energy (i) and intensity (ii) of each contribution as function of the NP diameter and refractive index of the surrounding medium.

### Small NP at low refractive index medium *vs* large NP at high refractive index medium

Au (**Fig. S.** 5) and Ag (**Fig. S.** 6) far- and near-field simulated spectra for 5 nm diameter NPs at $n$=1 and 100nm at $n$=2.

#### Au NP (S. 5)

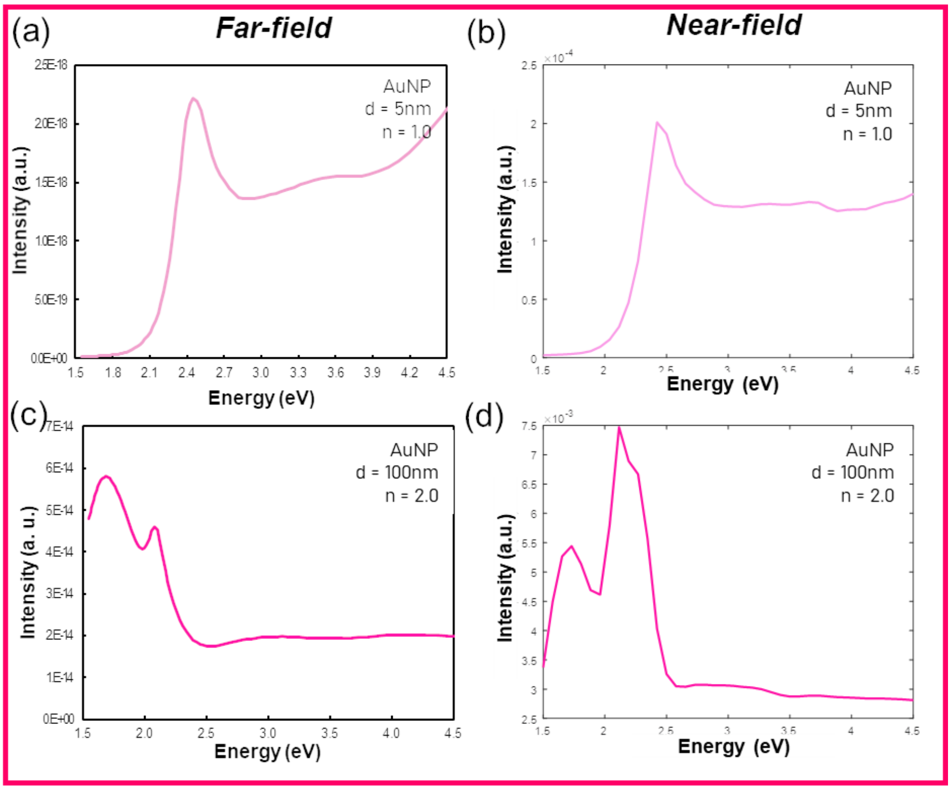


**S. 5:** Far- (a,c) and near-field (b,d) simulated AuNP spectra for the smaller diameter and lower refractive index (i. e., 5 nm diameter and n = 1) considered (a, b), and for the larger diameter and higher refractive index (i. e., 100 nm diameter and n = 2) regarded (c, d).

**Ag NP (S. 6)**

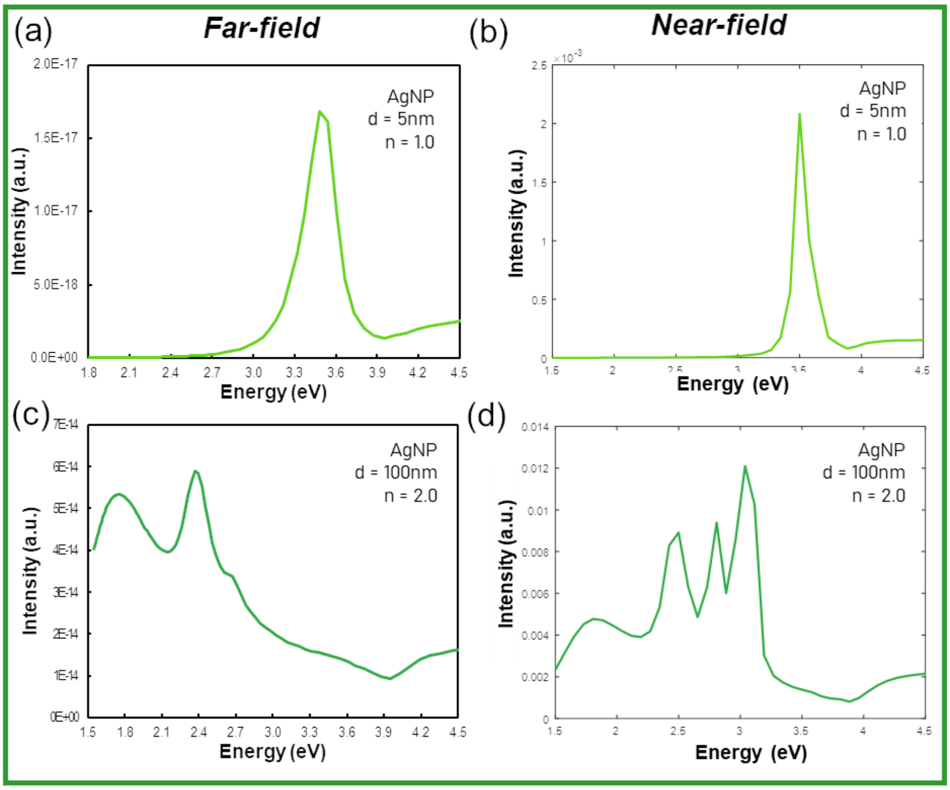


**S. 6:** Far- (a,c) and near-field (b,d) simulated AgNP spectra for the smaller diameter and lower refractive index (i. e., 5 nm diameter and n = 1) considered (a, b), and for the larger diameter and higher refractive index (i. e., 100 nm diameter and n = 2) regarded (c, d).

# References

[1] Low & ultra-low refractive index polymers.

[2] Tomoya Higashihara and Mitsuru Ueda. Recent progress in high refractive index polymers. *Macromolecules*, 48(7):1915–1929, 2015.

[3] Nicholas J. Wallace, Matthew R. Jones, and Nathan B. Crane. Spectral absorption coefficient of additive manufacturing materials. *Journal of Thermal Science and Engineering Applications*, 13(4), 01 2021. 041012.

[4] Angus W. Ritchie, Harrison J. Cox, Hassan I. Gonabadi, Steve J. Bull, and Jas Pal S. Badyal. Tunable high refractive index polymer hybrid and polymer–inorganic nanocomposite coatings. *ACS Applied Materials & Interfaces*, 13(28):33477–33484, 2021. PMID: 34254516.

[5] Wontae Jang, Keonwoo Choi, Ji Sung Choi, Do Heung Kim, Kookheon Char, Jeewoo Lim, and Sung Gap Im. Transparent, ultrahigh-refractive index polymer film (n $\approx$1.97) with minimal birefringence ($\delta$n $<$0.0010). *ACS Applied Materials & Interfaces*, 13(51):61629–61637, 2021. PMID: 34914349.

[6] Ulrich Hohenester and Andreas Trügler. Mnpbem - a matlab toolbox for the simulation of plasmonic nanoparticles. *Computer Physics Communications*, 183:370–381, 2 2012.

[7] Ulrich Hohenester. Simulating electron energy loss spectroscopy with the mnpbem toolbox. *Computer Physics Communications*, 185:1177–1187, 3 2014.
